# Preoperative Function Assessment of Ex Vivo Kidneys with Supervised Machine Learning Based on Blood and Urine Markers Measured during Normothermic Machine Perfusion

**DOI:** 10.3390/biomedicines10123055

**Published:** 2022-11-28

**Authors:** Wenke Markgraf, Hagen Malberg

**Affiliations:** Institute of Biomedical Engineering, Technische Universität Dresden, 01307 Dresden, Germany

**Keywords:** normothermic machine perfusion, organ preservation, kidney, function assessment, classification, supervised learning methods, urine marker, blood marker

## Abstract

Establishing an objective quality assessment of an organ prior to transplantation can help prevent unnecessary discard of the organ and reduce the probability of functional failure. In this regard, normothermic machine perfusion (NMP) offers new possibilities for organ evaluation. However, to date, few studies have addressed the identification of markers and analytical tools to determine graft quality. In this study, function and injury markers were measured in blood and urine during NMP of 26 porcine kidneys and correlated with ex vivo inulin clearance behavior. Significant differentiation of kidneys according to their function could be achieved by oxygen consumption, oxygen delivery, renal blood flow, arterial pressure, intrarenal resistance, kidney temperature, relative urea concentration, and urine production. In addition, classifications were accomplished with supervised learning methods and histological analysis to predict renal function ex vivo. Classificators (support vector machines, k-nearest-neighbor, logistic regression and naive bayes) based on relevant markers in urine and blood achieved 75% and 83% accuracy in the validation and test set, respectively. A correlation between histological damage and function could not be detected. The measurement of blood and urine markers provides information of preoperative renal quality, which can used in future to establish an objective quality assessment.

## 1. Introduction

The accomplishment of an adequate and objective assessment of renal graft quality in transplantation medicine is a long-known but unresolved problem. Progress in this regard could, among other things, avoid unnecessary rejection of transplants and expand the organ pool in the long term [1]. Consequently, more organs could be used for transplantation in the future, thus minimizing the currently existing organ deficit [1,2].

To date, there is no evaluation process that provides an accurate, evidence-based prediction of postoperative graft function. Thus, there is a lack of reliable methods to characterize organ status, which could assist the medical team in deciding whether to accept or reject an organ. Consequently, the final call on the use of an organ is usually based on a subjective decision relying on the experience of transplant physicians. [1]

In general, graft quality can be established at two stages of the transplant process: before procurement by evaluating donor-specific characteristics (e.g., Kidney Donor Risk Index) [3] and during static cold storage by performing pretransplant kidney biopsies (e.g., Remuzzi Score) [4]. However, these methods have limitations and their predictive value for determining postoperative renal function is uncertain [5,6].

Normothermic machine perfusion (NMP) during organ preservation offers new perspectives to transplantation medicine and a promising approach for objective organ evaluation. This method involves perfusing organs ex vivo with nutrient- and oxygen-containing, usually blood-based, solutions at body temperatures that restore the physiological milieu. Thus, the organ’s metabolism and function can be maintained outside the body [7]. Consequently, this allows the analysis of blood, urine, and tissue markers that can be evaluated for their suitability in predicting renal quality [8,9,10,11,12,13].

However, the development of strategies for ex vivo assessment of renal function remains poorly investigated. Optical imaging techniques such as hyperspectral imaging and magnetic resonance imaging were introduced to measure tissue characteristics and use them as a basis for future functional assessment [14,15]. Specifically, the studies examined ischemia-reperfusion injury and regional distribution of perfusate flow in the renal cortex. [14,15]. Encouraging primary results demonstrated the predictive potential for assessing postoperative organ function of the blood and urine markers: renal resistance, lactate clearance, acid-base homeostasis, oxygen consumption, neutrophil gelatin-associated lipocalin, endothelin-1, nanoparticles, and flavin mononucleotide [16,17,18,19]. In addition, human kidneys rejected for transplantation were assessed by the quality assessment score (QAS) based on macroscopic appearance, renal blood flow, and urine output [20,21,22]. The QAS correlates with DGF but not with long-term outcome, Kidney Donor Risk Index, or Remuzzi Score [16,21,22].

In our recently published study, we described the potential of preoperative renal function assessment by measuring inulin clearance, which is the gold standard for determining glomerular filtration rate in vivo and thus renal function [23]. Analysis of the ability of the kidneys to filter inulin during NMP allows evaluation of functional renal status ex vivo [23]. This may represent a promising approach for the development of new preoperative strategies to predict renal function, if postoperative renal function is also confirmed [23]. The use of hyperspectral imaging and KidneyResNet, a convolutional neural network (CNN) based on the ResNet-18 architecture, allowed noninvasive objective assessment of extracorporeal renal function in a laboratory porcine model based on tissue-related spectral properties [24]. Thus, ex vivo kidneys could be successfully differentiated into functional, limited functional, and nonfunctional [24]. The consequent next step is to investigate other markers, such as blood and urine markers, that are measurable without the use of a special imaging technology and thus more accessible to a wider range of researchers and clinicians, to determine their potential for predicting extracorporeal renal function.

The aim of the present work is the first functional classification of ex vivo kidneys with machine learning algorithms based on function and injury markers measured in blood and urine during NMP. Using various supervised learning methods, their potential for predicting the functional status of kidneys was evaluated. Here, inulin clearance served as a reference for ex vivo renal function. The results of the classifiers were compared with histologic analysis of Remuzzi Scores.

## 2. Materials and Methods

### 2.1. Study Design

The aim of this exploratory analysis was to perform functional classification of ex vivo kidneys by supervised learning methods based on blood and urine markers collected during organ preservation. For this purpose, blood and urine markers measured during a previously published study [23] were analyzed. In these experiments, the kidneys of healthy laboratory pigs were perfused normothermically for four hours. Ex vivo renal function was assessed by inulin clearance. To establish a function-based classification of kidneys, various classifiers and the preoperative Remuzzi Score were compared for their ability to predict renal quality.

### 2.2. Normothermic Machine Perfusion

**Figure 1:**
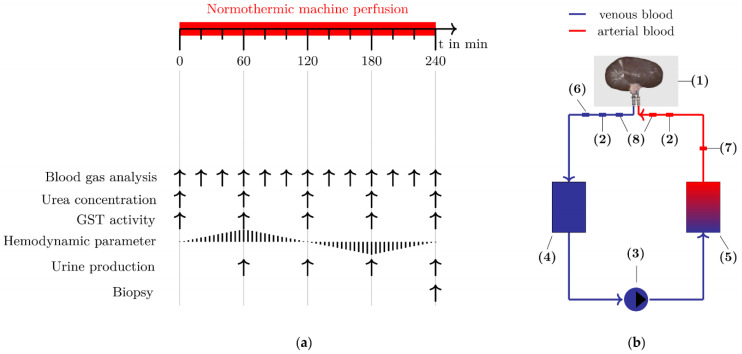
Normothermic machine perfusion (NMP) setup. (**a**) Before connecting the kidneys to the perfusion system, baseline blood samples were taken at t = 0 min. Subsequently, the blood, urine and tissue markers were measured at regular intervals. (**b**) The NMP system consists of the following components: (1) organ storage tray, (2) blood collection sites, (3) roller pump, (4) reservoir, (5) oxygenator, (6) temperature sensor, (7) pressure sensor, and (8) tube clamp.

Kidneys were preserved using a NMP method, which has been described in detail previously [23]. In brief, 28 kidneys were obtained from laboratory pigs after nonabdominal surgical training. Organs were exposed to different times of warm ischemia (4 min to 185 min) and then stored on ice (94 min to 790 min). Afterwards, the kidneys were preserved under near-physiological conditions for four hours. The perfusion solution used consisted of autologous whole blood continuously supplied with gas (0.5 L/min, 21% O_2_, 8–10% CO_2_, 69–71% N_2_), stabilized with a citrate-based solution, and added with glucose and NaHCO_3_ solutions. Blood and urine samples were collected at regular 20- and 60 min intervals during renal preservation (see Figure 1a). In addition, hemodynamic parameters were measured during NMP at discrete 0.2 s intervals. A schematic representation of the pressure-controlled perfusion system used is illustrated in Figure 1b.

### 2.3. Dataset

The dataset included blood, urine, and histologic data from kidneys preserved during our recently published NMP study [23]. Complete data could be collected from 26 kidneys. Two kidneys were excluded from the study because their perfusion had to be interrupted before 240 min had elapsed. The reason for discontinuation of the two NMP experiments were nonphysiological perfusion pressures (arterial pressure > 190 mmHg) in the NMP circuit that persisted for more than 30 min.

The ex vivo functional quality of kidneys from laboratory pigs was derived from GFR based on inulin excretion behavior and the percentage of inulin excreted from the blood during NMP. For detailed information on the measurement of inulin clearance and functional classification of the kidneys, the reader is referred to Markgraf et al. [23].

Three functional classes of kidneys could be identified [23]:

Class 1: nonfunctional kidneys (*n* = 4),

Class 2: limited functional kidneys (*n* = 10),

Class 3: functional kidneys (*n* = 12).

For classification, blood and urine data were labeled according to the functional status of each kidney. A stratified train-test split was performed. 77% (20 kidneys) were used as training and validation data and 23% as test data (*n* = 6).

Further details on the characteristics of all kidneys in each functional class, kidneys in the test data, and training and validation data can be found in the Supplementary Material (see Table S1: Characteristics of all kidneys).

### 2.4. Function Markers for Metabolic Activity

The parameters required for the calculation of the function markers of metabolic activity were measured with a blood gas analyzer (ABL 80 FLEX BASIC, Radiometer GmbH, Willich, Germany) at defined time (see Figure 1a). All function markers listed in this subchapter are normalized to a kidney weight of 100 g.

#### 2.4.1. Oxygen Delivery

The oxygen delivery DO_2_ (in mL/min) describes the amount of oxygen supplied to the tissue in each minute, where RBF represents the renal blood flow in mL/min and C_A_O_2_ the oxygen content of arterial blood in mL/dL (see Equation (1)) [25].

(1) $${\mathrm{DO}}_{2}=\mathrm{RBF}\;\cdot {\;C}_{A}O_{2}\;,$$

C_A_O_2_ is the sum of oxygen bound to hemoglobin and oxygen dissolved in plasma (see Equation (2), modified from [26]), where 1.34 is the Hüfner constante in mL, c(Hb) is the median hemoglobin concentration in g/dL of all times of measurement during NMP of one kidney, sO_2_ is the oxygen saturation in %, 0.0031 the solubility coefficient of oxygen in plasma in mL/(mmHg∙dL), and pO_2_ is the partial pressure of oxygen in mmHg.

(2) $$C_{A}O_{2}=1.34\;\cdot \;c\left(\mathrm{Hb}\right)\cdot \frac{{\mathrm{sO}}_{2}}{100}+0.0031\;\cdot {\mathrm{pO}}_{2}\;,$$

#### 2.4.2. Oxygen Consumption

Oxygen consumption $\dot{V}O_{2}$ (in mL/min) is the volume of oxygen consumed by the tissue per minute. This marker was calculated using Fick’s principle as follows (see Equation (3), [25]):

(3) $$\dot{V}O_{2}=\mathrm{RBF}\;\cdot \;(C_{A}O_{2}-{\;C}_{V}O_{2})\;,$$

Here, C_V_O_2_ represents the oxygen content of venous blood in mL/dL and is determined from the corresponding parameters of the venous blood according to Equation (2).

#### 2.4.3. Oxygen Extraction

The oxygen extraction ERO_2_ (in %) is the proportion of oxygen supplied via the blood, that is consumed by tissues. Thus, ERO_2_ is the ratio of oxygen consumption to oxygen delivery (see Equation (4)):

(4) $${\mathrm{ERO}}_{2}=\frac{\dot{V}O_{2}}{{\mathrm{DO}}_{2}}\;,$$

#### 2.4.4. Carbon Dioxide Production

Carbon dioxide production CO_2_P (in L/min) is the product of the RBF and the arterio-venous difference in carbon dioxide concentration (see Equation (5), [27]), where C_V_CO_2_ represents the venous carbon dioxide concentration in mL/L and C_A_CO_2_ the arterial carbon dioxide concentration in mL/L.

(5) $${\mathrm{CO}}_{2}P=\mathrm{RBF}\;\cdot \;(C_{V}{\mathrm{CO}}_{2}-{\;C}_{A}{\mathrm{CO}}_{2})\;,$$

For a detailed description of the calculation of arterial or venous carbon dioxide concentration, please refer to the literature of Boode et al. and Douglas et al. [27,28].

### 2.5. Function Markers for Renal Hemodynamics

The sensors for measuring arterial pressure (AP) and kidney temperature (T_Kidney_) are integrated into the NMP system (see Figure 1b, (6), (7)). The RBF results from the pumping speed of the roller pump (see Figure 1b, (3)). The intrarenal resistance IRR (in mmHg/(mL/min)) is the quotient of AP and RBF (see Equation (6)):

(6) $$\mathrm{IRR}=\frac{\mathrm{AP}}{\mathrm{RBF}}\;,\;$$

T_Kidney_, AP and RBF were recorded at discrete 0.2 s intervals during the NMP. Hemodynamic parameters, however, are analyzed at regular 20 min intervals. The value of a hemodynamic marker at an exact analysis time resulted from the mean value of the measured values in the time span: analysis time ± 2 min.

RBF and IRR were normalized to a kidney weight of 100 g.

The setting of hemodynamic parameters for NMP of ex vivo kidneys was performed as follows: After the kidneys were connected to the NMP circuit, a systemic RBF of 50 mL/min was set for the first few minutes to check for AP in the system. If the AP was within the defined physiological range (AP < 110 mmHg), the pressure was set to an AP = 100–110 mmHg and the RBF was no longer changed manually. When the AP was > 110 mmHg, the RBF was reduced manually until the AP reached constant values.

### 2.6. Function Markers for Renal Filtration

#### 2.6.1. Urea Concentration

Blood samples were collected at defined times (see Figure 1a). After centrifugation at 4 °C and 10,000× *g* for 15 min, the supernatant was obtained and stored at −30 °C. Quantitative analysis of urea in blood plasma was performed using an urea assay kit (KA1652, Abnova GmbH, Heidelberg, Germany). The marker was normalized to a kidney weight of 100 g.

#### 2.6.2. Urine Production

The urine was collected in a sterile tube throughout the NMP experiment. The volume of urine produced was measured at specific times (see Figure 1a). The marker was normalized to a kidney weight of 100 g.

### 2.7. Injury Marker for Tubular Impairment

During the NMP experiments, blood was collected from the NMP circuit at regular times for glutathione S-transferase (GST) activity analysis (see Figure 1a). After centrifugation for 15 min at 4 °C and 10,000× *g*, the supernatant was obtained and stored at −30 °C. Quantitative analysis of GST activity in blood plasma was performed using a GST assay kit (CS0410, Sigma Aldrich GmbH, Steinheim, Germany).

### 2.8. Supervised Learning Methods for the Functional Classification of Ex Vivo Kidneys

#### 2.8.1. Testing for Multicollinearity

Before using the blood and urine markers as input for the classifiers, their multicollinearity had to be excluded. Highly correlated markers had to be eliminated. Multicollinearity could be determined by the variance inflation factor (VIF), which was calculated from the diagonal elements of the inverse of the correlation matrix [29]. In Matlab (Matlab 2018b, The MathWorks, Inc., Natick, MA, USA), VIF could be calculated as follows:

(7) VIF= diag(corrcoef(x))’ 

If VIF > 10, the respective maximum of the interdependent variables is removed from the data set and the multicollinearity test is reapplied to check whether acceptable VIF (VIF ≤ 10) are finally achieved.

#### 2.8.2. Supervised Learning Methods

In this study, the following most widely used supervised learning methods were investigated for their suitability for functional kidney classification: support vector machine (SVM), random forest (RF), k-nearest-neighbor (kNN), logistic regression (LOG), and naive bayes (BAY). A detailed description of the principles of the methods is described in [30], among others. Classifiers were implemented in python (python 3.8.8, Python Software Foundation, Wilmington, DE, USA) using the *sklearn* and *numpy libraries*.

The implementation of the SVM was realized with the *svm.SVC* function using a linear kernel and the one-vs-one strategy.

The RF was implemented using the *RandomForestClassifier*. The number of decision trees was 20.

The *KNeighborsClassifier* function was used for implementation of the kNN classifier. The number of neighbors, which are all equally weighted, was 5. The Euclidean distance was considered as distance measure.

The LOG was implemented using the *LogisticRegression* function.

The BAY algorithm was implemented using the *GaussianNB* function.

#### 2.8.3. Validation

The kidneys used for classification were divided into a training and validation dataset and a test dataset using the holdout method (see Supplementary Material: Table S1: Characteristics of all kidneys). Due to the overall small dataset, leave-one-out cross-validation was used to estimate the classification quality for validation. Each individual kidney with all corresponding markers at all times of measurement was used once as a validation dataset. However, leave-one-out cross-validation was only applied during the optimization process. For the final model, the optimized parameters were used to retrain the model incorporating all training data without validation split. This final prediction model was applied to the hold-out test dataset. The classification quality was estimated using accuracy, precision and recall. Here, accuracy describes the ratio of the sum of all kidneys assigned as true negatives and true positives to the total number of classified kidneys. Precision is to the quotient of kidneys assigned as true positives across all classes and the total number of kidneys assigned as true positives and false positives across all classes. Recall corresponds the ratio of kidneys assigned as true positives across all classes to the total number of kidneys assigned as true positives and false negatives across all classes.

Accuracy was implemented via the *cross_val_score* function. Precision was calculated via the *metrics.precision_score* function and recall via the *metrics.recall_score* function both with average = ‘minor’.

### 2.9. Histological Assessment Using the Preoperative Remuzzi Score

After four hours of NMP treatment, a biopsy was taken from the upper pole of all kidneys (see Figure 1a). Histological specimens were fixed in formalin and stained with Masson-Goldner staining. A blinded histopathological evaluation of the biopsies was performed by an experienced pathologist according to the Remuzzi Score [4].

### 2.10. Statistical Analysis

Data normality and homoscedasticity were verified using the Shapiro–Wilk [31] and Levene’s test [32]. If homoscedasticity was violated in normally distributed data (*p* ≤ 0.05), a Welsh ANOVA [33] following a Games-Howell post hoc test [34] was applied to evaluate significant differences between the groups. If homoscedasticity was assumed in normally distributed data (*p* > 0.05), an ANOVA following Bonferroni post hoc test was performed. For a non-normal distribution, a Kruskal–Wallis-Test following a Dunn’s-Sidak post hoc test was used. *p* ≤ 0.05 was considered significant. Matlab 2018b was used for statistical analysis.

## 3. Results

### 3.1. Function Markers for Metabolic Activity

**Figure 2:**
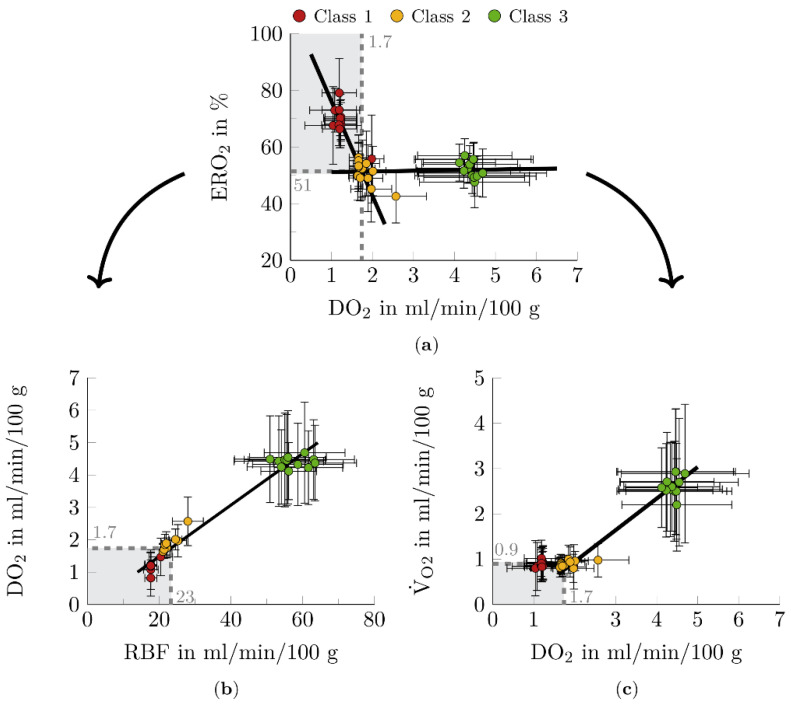
Function markers for metabolic activity. (**a**) Dependence oxygen extraction (ERO_2_) on oxygen delivery (DO_2_). At a critical point (DO_2, crit_ = 1.7 mL/min/100 g, ERO_2, crit_ = 51%) corresponding to the intersection of the two lines (f(x)_1_ = −33.337x + 109.43, f(x)_2_ = 0.2292x + 50.933; shown as black lines), ERO_2_ increased considerably. (**b**) Dependence DO_2_ on renal blood flow (RBF). The data suggest a linear relationship between the RBF and DO_2_ (R^2^ = 0.97, y = 12.57x + 1.3984; shown as black line). RBF_crit_ was determined by substituting DO_2, crit_ into the linear equation. (**c**) Dependence of oxygen consumption ($\dot{V}O_{2}$) on DO_2_. When the critical point (DO_2, crit_ = 1.7 mL/min/100 g, $\dot{V}O_{2,\;\mathrm{crit}}$ = 0.9 mL/min/100 g) was exceeded, the amount of oxygen consumed by the tissue increased. Considering the linear behavior shown in (**a**), the relationship between DO_2_ and $\dot{V}O_{2}$ could be described as quadratic below the critical point (y = −0.696 × 2 + 1.7915x − 0.3326, shown as black curve), and linear beyond (R^2^ = 0.94, y = 0.696x − 0.4451, shown as black line). The gray areas illustrate the critical areas of the function markers. Data points represent the twelve times of measurement during the NMP. Plot mean ± SEM. *n* = 3 for nonfunctional kidneys (class 1), *n* = 5–7 for limited functional kidneys (class 2), *n* = 9–11 for functional kidneys (class 3). *n* < actual number of kidneys per class (see Section 2.3), because unfortunately blood gas analysis could not always be performed for all kidneys at all time points.

**Figure 3:**
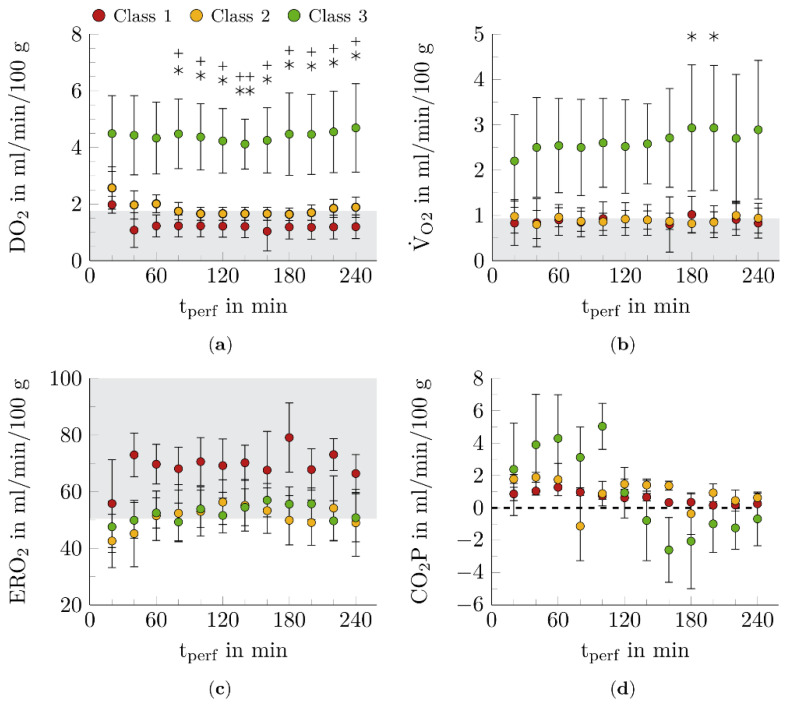
Course of function markers for metabolic activity during NMP. The perfused kidneys showed a correlation between the predicted renal function class and the function markers (**a**) DO_2_, (**b**) $\dot{V}O_{2}$, (**c**) ERO_2_, and (**d**) carbon dioxide production (CO_2_P). The gray areas illustrate the critical regions of the function markers. The data points represent the twelve times of measurement during the NMP. Shown mean ± SEM. *n* = 3 for nonfunctional kidneys (class 1), *n* = 5–7 for limited functional kidneys (class 2), *n* = 9–11 for functional kidneys (class 3). *, *p* < 0.05, **, *p* < 0.01, class 2 vs. class 3; +, *p* < 0.05, ++, *p* < 0.01, class 1 vs. class 3. *n* < actual number of kidneys per class (see Section 2.3), because unfortunately blood gas analysis could not always be performed for all kidneys at all time points. During NMP, function markers DO_2_, $\dot{V}O_{2}$, ERO_2_, and CO_2_P varied as a function of renal functional class and perfusion time (t_perf_) (see Figure 3).

In this study, four functional markers of metabolic activity during NMP were determined (see Figure 2 and Figure 3).

To meet the metabolic energy demands of the ex vivo organ to maintain renal function, ERO_2_ initially remained approximately constant as DO_2_ decreased (see Figure 2a). After reaching a critical point at DO_2, crit_ = 1.7 mL/min/100 g and ERO_2, crit_ = 51%, a steep increase in ERO_2_ with further decrease in DO_2_ was demonstrated (see Figure 2a). This behavior may indicate a deficiency of oxygen.

To ensure adequate oxygen supply to the kidneys, other parameters related to DO_2_ are considered for the analysis. To guarantee the transport of an adequate amount of oxygen to the tissues, renal blood flow must not fall below RBF_crit_ = 23 mL/min/100 g (see Figure 2b). In addition, the amount of oxygen consumed by the tissue is not assumed to be sufficient to meet the metabolic energy demands of the organ until a $\dot{V}O_{2,\;\mathrm{crit}}$ = 0.9 mL/min/100 g (see Figure 2c).

DO_2_ reached the highest value in class 3 kidneys and remained approximately constant during NMP (see Figure 3a). In class 1 and 2 kidneys, DO_2_ decreased during the first hour and then remained stable. The DO_2_ of class 1 and 2 kidneys fell below the critical threshold DO_2, crit_ = 1.7 mL/min/100 g, after 40 and 100 min, respectively. From 80 min NMP, kidneys of class 3 could be significantly differentiated from kidneys of the other two classes (*p* < 0.05, *p* < 0.01).

In comparison, $\dot{V}O_{2}$ increased in class 3 kidneys during the four-hour NMP (see Figure 3b). Whereas class 1 and 2 kidneys consumed constant amounts of oxygen that were predominantly less than $\dot{V}O_{2,\;\mathrm{crit}}$. In general, class 3 kidneys showed higher $\dot{V}O_{2}$, which was significantly different from class 2 at 180 min and 200 min perfusion time (*p* < 0.05).

ERO_2_ was higher in class 1 kidneys than in class 2 and 3 kidneys (see Figure 3c). However, this difference was not significant.

Finally, CO_2_P was considered (see Figure 3d). At the beginning of NMP, CO_2_P increased, which was most pronounced in class 3 kidneys. These kidneys showed a transition from carbon dioxide production to carbon dioxide conversion after 120 min of NMP.

### 3.2. Function Markers for Renal Hemodynamics

**Figure 4:**
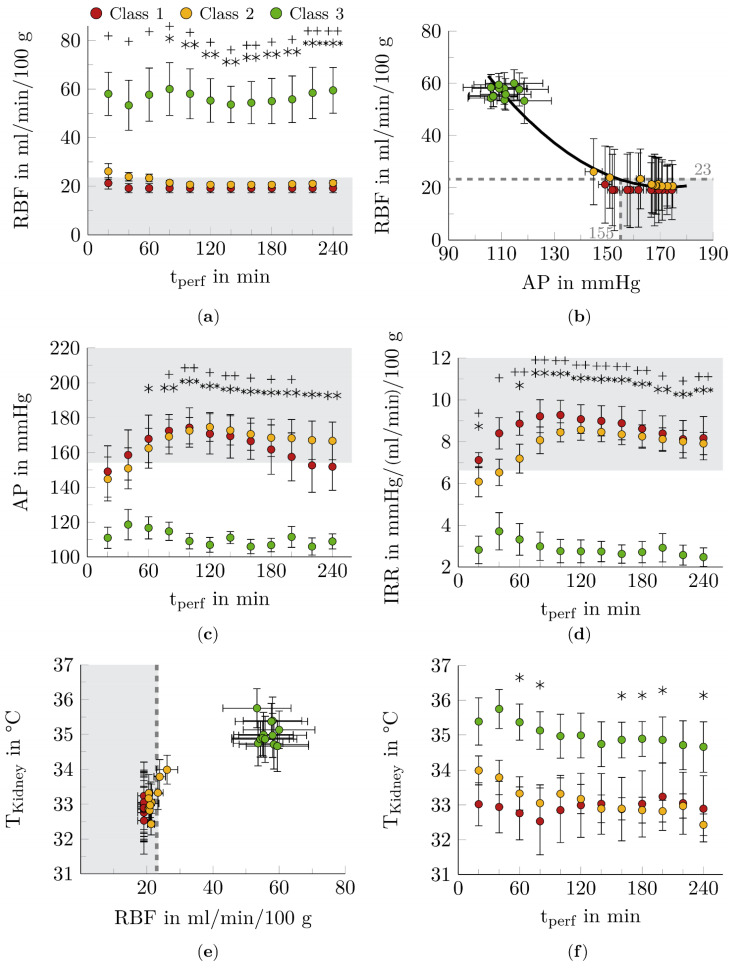
Course of function markers for renal hemodynamic during NMP. A dependency between the predicted functional class and the function markers for hemodynamics could be demonstrated. Kidneys of class 3 behaved significantly different from kidneys of the other two classes regarding renal blood flow (RBF) (**a**), arterial pressure (AP) (**c**) and intrarenal resistance (IRR) (**d**). (**b**) The relationship between AP and RBF was described using a 2nd degree polynomial function (R^2^ = 0.96, y = 0.0093x^2^ − 3.2127x + 297.6, shown as a black curve). The dependence of T_Kidney_ on RBF (**e**) and kidney function (**f**) was shown. The gray areas illustrate the critical regions of the function markers. The data points represent the twelve times of measurement during the NMP. Shown mean ±SEM. *n* = 4 for nonfunctional kidneys (class 1), *n* = 10 for limited functional kidneys (class 2), *n* = 12 for functional kidneys (class 3), *, *p* < 0.05, **, *p* < 0.01, ***, *p* < 0.001, class 2 vs. class 3; +, *p* < 0.05, ++, *p* < 0.01, class 1 vs. class 3.

Four functional markers of renal hemodynamics were examined for their correlation with ex vivo renal function (see Figure 4).

Class 3 kidneys showed the highest RBF over the entire NMP period (see Figure 4a). After 20 and 80 min of NMP treatment, these kidneys could be clearly distinguished from class 1 and class 2 kidneys, respectively. RBF of the kidneys of classes 1 and 2 fell below the RBF_crit_ during NMP.

Based on the renal autoregulatory capacity, a correlation between AP and RBF was expected in normothermically perfused kidneys (see Figure 4b). There seems to be a trend for RBF to decrease steadily with increasing AP, reaching AP_crit_ = 155 mmHg with RBF_crit_ = 23 mL/min/100 g. A further increase in AP above AP_crit_ resulted in only a slight decrease in RBF.

In general, ex vivo perfused kidneys showed an increase in AP at the beginning of NMP (see Figure 4c). The AP decreased again during NMP, and an approximately constant course was obtained for class 3 kidneys after 80 min, followed by class 2 kidneys after 160 min and class 1 after 220 min. During NMP, kidneys of classes 1 and 2 reached AP_crit_ after 60 min. At this time, significant differences between kidneys of class 3 and those of the other two classes were detected for the first time (*p* < 0.05-*p* < 0.001).

Using IRR, a significant difference between class 3 kidneys and class 1 (*p* < 0.05-*p* < 0.01) and class 2 (*p* < 0.01-*p* < 0.001) kidneys was observed already at the beginning of NMP (see Figure 4d).

The T_Kidney_ depends on the RBF during perfusion (see Figure 4e). The lower the RBF, the lower the T_Kidney_. If the RBF_crit_ = 23 mL/min/100 g was not exceeded, the kidneys were not supplied with physiologically tempered blood and perfusion of the kidneys under normothermic conditions could not be ensured. Class 1 kidneys had temperatures below 33.5 °C throughout the NMP (see Figure 4f). Class 2 kidneys showed a similarly low temperature after 120 min of NMP. Kidneys assigned to the function class 3 showed permanent normothermic T_Kidney_ values.

### 3.3. Function Markers for Renal Filtration

**Figure 5:**
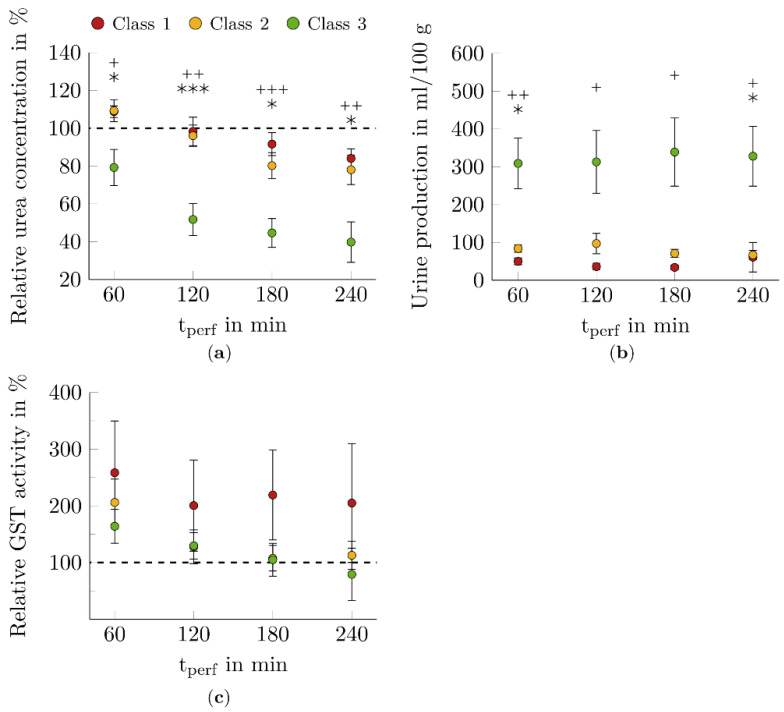
Course of markers for renal filtration and tubular impairment during NMP. The perfused kidneys showed a dependence between the predicted functional class and the function markers (**a**) relative urea concentration and (**b**) urine production. The relative glutathione S-transferase (GST) activity varies depending on the renal function (**c**). However, no significant differentiation of the kidneys could be observed. The dashed lines correspond to (**a**) relative urea concentration and (**b**) relative GST activity of 100% at t_0_. The data points represent the twelve times of measurement during the NMP. Shown mean ± SEM. *n* = 4 for nonfunctional kidneys (class 1), *n* = 10 for limited functional kidneys (class 2), *n* = 12 for functional kidneys (class 3), *, *p* < 0.05, ***, *p* < 0.001, class 2 vs. class 3; +, *p* < 0.05, ++, *p* < 0.01, +++, *p* < 0.001, class 1 vs. class 3.

Function markers investigated for renal filtration were the relative urea concentration in the blood and the amount of urine produced during NMP (see Figure 5a,b).

Blood urea concentration decreased during NMP (see Figure 5a). Thereby, class 3 kidneys excreted significantly higher amounts of urea compared to kidneys of class 1 (*p* < 0.05-*p* < 0.001) and class 2 (*p* < 0.05-*p* < 0.001).

Hourly urine output of class 3 kidneys was consistently high and significantly different from class 1 (*p* < 0.05-*p* < 0.01) and class 2 (*p* < 0.05) kidneys (see Figure 5b).

### 3.4. Injury Marker for Tubular Impairment

As part of the study, GST activity was determined in plasma (see Figure 5c). The relative GST activity initially increased in all kidneys. The increase was most pronounced in class 1 kidneys. These kidneys showed approximately constant high values during the further course of NMP. In class 2 and class 3 kidneys, GST activity decreased continuously and returned to baseline values after four hours of NMP.

### 3.5. Detection of Multicollinearity

To enable classification of kidneys using supervised learning classifiers, multicollinearity among the blood and urine markers had to be excluded. Markers that are strongly correlated with each other were identified using VIF (see Supplementary Material Table S2: Testing for multicollinearity).

Considering all 11 markers, two markers (RBF, AP) showed high correlation and three markers (IRR, $\dot{V}O_{2}$, DO_2_) showed critical correlation. After excluding the IRR as well as the DO_2_, a moderate correlation of the other markers could be determined. These results allow the following classification based on the 9 remaining markers.

### 3.6. Functional Classification Based on Blood and Urine Markers

Five classifiers were used to test the classification of kidneys into three functional classes: nonfunctional (class 1), limited functional (class 2), and functional (class 3).

Subsequently, it was investigated which of the five classifiers yielded the best possible results when applying the 9 markers and values of 4 or 12 measurement times. The algorithms were executed in both the validation data set and the test data set for the classification of the kidneys. In the following, a specific measurement times of a marker is referred to as a feature. Five variants were tested, differing in the number of features used as input to the classifiers. These variants considered all parameters for which significant differences between classes were found, including all times of measurement up to 240 min (variant A, 56 features) and markers and their times of measurement up to 240 min (variant B, 38 features), 180 min (variant C, 27 features), 120 min (variant D, 16 features), and 60 min (variant E, 7 features) at which significant differences between classes occurred.

An NMP up to and including 180 min with acquisition of the significant markers RBF, AP, T_kidney_, $\dot{V}O_{2}$, urine production and relative urea concentration was necessary to obtain the best results using the classifiers SVM, kNN, LOG and BAY on both the validation (see Supplementary Material Table S3: Functional classification based on blood and urine markers, variant C) and the test dataset (see Supplementary Material Table S4: Functional classification based on blood and urine markers, variant C). On the validation dataset, values of 0.75 were achieved for accuracy, precision and recall. In contrast, an accuracy of 0.83, a precision of 0.72 and a recall of 0.83 were achieved on the test dataset.

**Figure 6:**
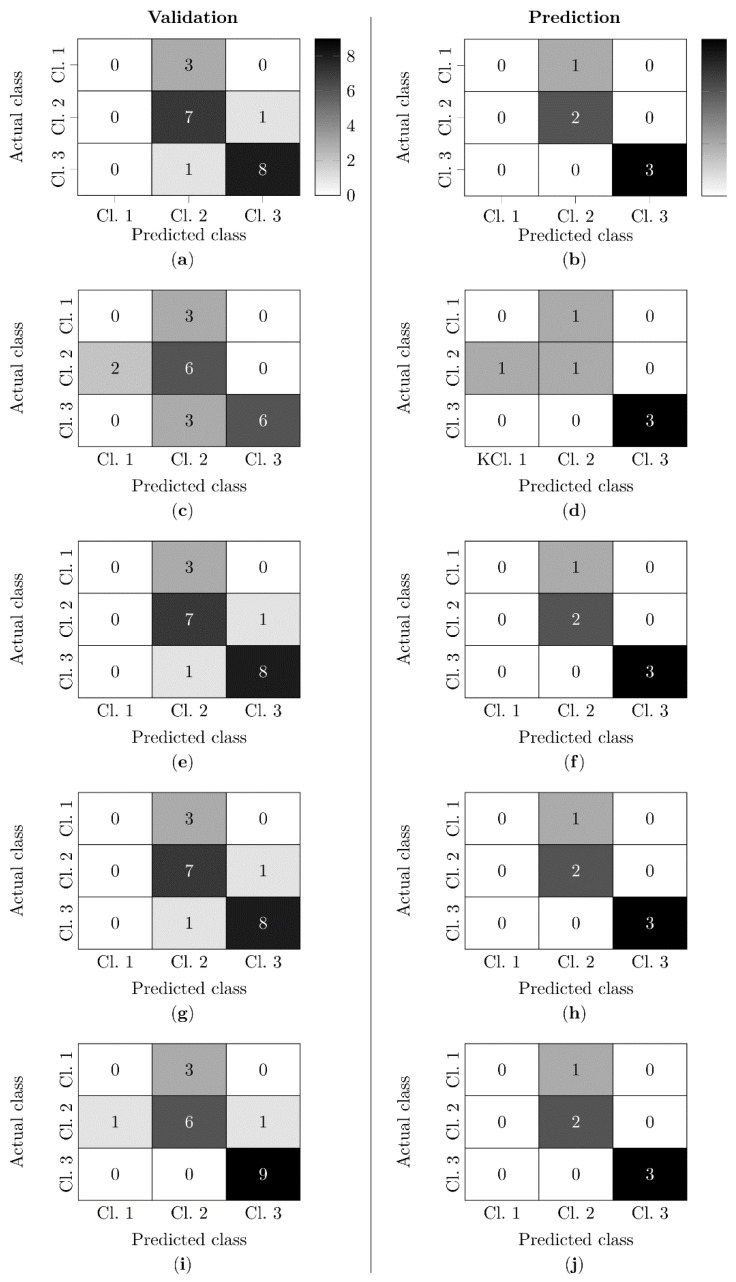
Confusion matrices for the classification of kidneys into three classes. Results of the classifiers for validation/prediction: support vector machine (SVM) (**a**,**b**), random forest (RF) (**c**,**d**), k-nearest-neighbor (kNN) (**e**,**f**), logistic regression (LOG) (**g**,**h**), naive bayes (BAY) (**i**,**j**).

In the confusion matrices, the prediction of the kidneys into the corresponding functional class was shown (see Figure 6). For the SVM, LOG and kNN, an identical partitioning of the kidneys was demonstrated. The BAY classifier was the only one able to correctly classify all functional kidneys (class 3) in the validation dataset. RF achieved the worst results in both validation and prediction.

An overview of all kidneys shows that the majority of kidneys could be assigned to a correct functional class based on the 27 features (see Table 1). An exception was the limited functional kidney 6, which could not be correctly classified by any of the classifiers. In addition, all nonfunctional kidneys (17, 21, 22, 25) could not be classified correctly.

### 3.7. Classification Based on the Preoperative Remuzzi Score

The normothermically perfused kidneys consistently exhibited mild to moderate histological injury based on the overall Remuzzi Score (see Supplementary Material Figure S1: Classification based on the preoperative Remuzzi Score). Furthermore, if only tubular atrophy, the parameter of the Remuzzi score that can detect acute injury (see Supplementary Material Table S5: Classification based on the tubular atrophy), is considered, no relation with renal function was found.

In conclusion, a correlation between histological damage and ex vivo function could not be detected.

## 4. Discussion

The present work is the first to classify normothermically perfused kidneys according to their ex vivo functional status using supervised machine learning algorithms based on blood and urine markers. Significant differentiation of kidneys according to their function could be achieved by eight-$\dot{V}O_{2}$, DO_2_, RBF, AP, IRR, T_Kidney_, relative urea concentration and urine production-of the eleven function and injury markers studied. However, based on supervised learning methods and using only the markers RBF, AP, T_kidney_, $\dot{V}O_{2}$, urine production and relative urea concentration, the classification between functional kidneys from kidneys with impaired function could be ensured. These methods can thus form the basis for the development of new diagnostic tools for the non-invasive assessment of organs.

### 4.1. Function Markers for Metabolic Activity

Of the four function markers of metabolic activity considered, $\dot{V}O_{2}$ and DO_2_, but not ERO_2_ and CO_2_P, varied significantly with renal function (see Figure 3).

The determination of $\dot{V}O_{2}$ has been performed in numerous preclinical and clinical NMP studies, which have focused on therapeutic strategies [35,36,37,38,39,40,41], the ideal NMP experimental setup [42,43,44,45,46,47], or the performance of NMP compared to other preservation methods [48,49,50], among others. In these studies, either Fick’s principle (e.g., [35,42]) or the simplified form, the arteriovenous pO_2_ difference without consideration of hemoglobin concentration (e.g., [37,39]), served as the basis for $\dot{V}O_{2}$ calculation.

A correlation between the $\dot{V}O_{2}$ and the QAS was previously investigated in three studies [16,51,52]. The level of $\dot{V}O_{2}$ was significantly higher in kidneys that were candidates for transplantation (score 1–2) than in kidneys that were no longer suitable as grafts (score 3–5) [16,51]. Moreover, this parameter was associated with a low donor creatinine level before organ retrieval [16]. One study also investigated the dependence of ERO_2_ on QAS. During NMP, a higher ERO_2_ value was associated with a higher QAS value [16].

The parameters DO_2_ and CO_2_P, which were also investigated in the present work, have not yet been used for functional assessment in other studies. In particular, DO_2_ showed promising results (see Figure 3a) and should be investigated in future work.

Not only the dependencies between function markers of metabolic activity and renal quality determined in the present work, but also the magnitude of their measured values are supported by the literature. The measured values for $\dot{V}O_{2}$, DO_2_, and ERO_2_ already published in the literature could be confirmed. Kidneys perfused with autologous whole blood for three hours after 10 min WIT and 17 h CIT, and thus conditioned similarly to functional class 3 kidneys, showed a $\dot{V}O_{2}$ = (2.0 ± 0.8) mL/min/100 g, an ERO_2_ = 60% ± 2%, and a DO_2_ = (4.4 ± 2.4) mL/min/100 g [42]. In comparison, this work demonstrated for functional kidneys a $\dot{V}O_{2}$ = (2.6 ± 0.3) mL/min/100 g, an ERO_2_ = 52 ± 2 %, and a DO_2_ = (4.4 ± 0.4) mL/min/100 g.

### 4.2. Function Markers for Renal Hemodynamics

The function markers RBF, AP, IRR, and T_Kidney_ showed significant differences between functional and limited functional or nonfunctional kidneys (see Figure 4). In the context of HMP, the predictive value of hemodynamic data for organ outcome has been part of intensive investigations. In a large randomized controlled trial, IRR at the end of HMP was shown to be an independent risk factor for both delayed graft function and 1-year graft failure [53]. No specific correlations could be found for the markers RBF and AP.

In studies with research focus on NMP, the hemodynamic perfusion markers RBF, AP, and IRR were commonly measured. In a large animal study in which kidneys were preserved during eight hours of NMP, kidneys not exposed to WIT had the lowest IRR, followed by kidneys exposed to 30 and 60 min of WIT [17]. Furthermore, IRR at the beginning of NMP correlated with postoperative renal function [17].

Relationships between the hemodynamic parameter RBF and WIT were determined in a preclinical study. RBF after 60 min of NMP was (47.2 ± 6.5) mL/min/100 g (7 min WIT), (33.2 ± 5.6) mL/min/100 g (15 min WIT), (31.5 ± 6.0) mL/min/100 g (25 min WIT), and (25.6 ± 4.7) mL/min/100 g (40 min WIT) and remained nearly constant during reperfusion up to six hours for all WIT [54]. Nevertheless, RBF was significantly decreased with 40 min warm ischemia compared with seven minutes warm ischemia after one- and six-hours reperfusion [54].

The dependence of WIT on renal hemodynamics and thus on functional impairment induced is confirmed in this work. The only currently existing scoring system for the assessment of renal quality, the QAS, includes the RBF as one of the three assessment criteria [20]. A large multicenter randomized controlled trial is currently underway to confirm the results between the QAS and renal quality [55].

In this study, we did not work with the commonly used arterial pressure of AP = 75 mmHg but set pressures of 100–110 mmHg for kidneys in which AP adaptation was possible [45,56]. There are two reasons for this. First, AP of >75 mmHg in whole blood perfused kidneys showed better overall renal function [57]. Second, physiological AP > 75 mmHg for pigs of different body weights have been reported in the literature [58].

With the AP chosen (100–110 mmHg), it was possible to ensure an NMP of functional kidneys [23]. However, we are aware that other arterial pressures will have to be tested in subsequent studies to ensure optimal perfusion of ex vivo kidneys.

### 4.3. Function Markers for Renal Filtration

The function markers of renal filtration investigated in this work were urine output and blood urea concentration. Both markers varied significantly between functional and limited functional/nonfunctional kidneys during NMP (see Figure 5).

Urine production is a criterion of QAS [20]. In addition, a preclinical study determined the dependence between WIT and urine production. Kidneys with a 40 min WIT had significantly lower urine output than kidneys with a 10 min WIT [59].

Blood urea concentration is a retention marker for kidneys and has not been determined in any NMP study to date. However, blood urea nitrogen derived from blood urea concentration was analyzed postoperatively after eight hours of NMP by Selzner et al. [17]. The study showed that the nitrogen content of urea in blood differed significantly between kidneys with WIT of 0 min, 30 min, and 60 min [17].

### 4.4. Injury Marker for Tubular Impairment

GST was found to be significantly associated with allograft outcomes in HMP studies. Associations with GFR and primary nonfunction were found in most prospective studies. [60]

To date, GST has been investigated in only one other study of NMP. Here, however, the isoenzyme GSTA1 was measured in the serum of patients after transplantation of a previously normothermically perfused kidney [61]. Thus, a comparison with the results collected in this paper is not possible. Tubular damage was higher in kidneys assigned as nonfunctional. However, no significant relationship with renal function was observed (see Figure 5).

### 4.5. Functional Classification Based on Blood and Urine Markers

In the present work, inulin clearance, the clinical gold standard for the assessment of renal function, is used as the basis for classification. Function markers of metabolic activity ($\dot{V}O_{2}$), renal hemodynamics (RBF, AP, T_Kidney_) and renal filtration (urine production, relative urea concentration) could be identified as suitable markers that correlate with inulin clearance and thus allow a functional classification of the kidneys (see Supplementary Material Table S3/S4: Functional classification based on blood and urine markers). It should be emphasized that an NMP of three hours is decisive for the assessment of the organs. Blood and urine markers used as input for the supervised learning method algorithms have been used in other NMP studies for the assessment of renal quality [16,17,18,19,20,21,22]. The novelty of our study is the classification of kidneys using algorithms of supervised learning methods based on these markers with ex vivo inulin clearance as reference parameter. Using the SVM, kNN, LOG, and BAY classifiers, correct prediction of function was achieved for 15 of 20 kidneys studied in the training dataset and for 5 of 6 kidneys in the test dataset (see Figure 6, Table 1). However, none of the classifiers could adequately classify nonfunctional kidneys. A comparable study is not known in the literature.

Working groups that focused on organ evaluation in NMP postulate that only a combination of different markers can provide an appropriate quality assessment of kidneys [14,15,16,17]. For their part, these research groups are attempting a functional assessment based on blood and urine markers. This statement can be confirmed by the results shown in this work. No single blood or urine marker could realize a functional classification.

However, a recent study developed a novel evaluation strategy based solely on VIS/NIR spectroscopic tissue properties that achieved better classification results for the dataset that was also used in this study [24]. Using HSI in combination with an appropriate convolutional neural network model, it was possible to predict renal quality during NMP and to classify kidneys into three functional classes [24].

Classification based on urine and blood markers offers potential for optimization. In addition to optimization of supervised learning methods, expanding the dataset is a possible approach for further research.

Optimization of the classifiers used in this work (SVM, LOG, RF, kNN and BAY) might improve the classification result. The supervised learning methods were implemented so that they can be used for ex vivo kidney evaluation. However, a systematic adaption of the classifiers by variation of the hyperparameters with comparison of the classification results was not carried out and should be the focus of further research. In addition, the combination of several machine learning models can also be explored to improve the classification results.

Larger datasets might help classifiers to better generalize to new data. Due to the small amount of data in the present work and the resulting limited number of training (20 kidneys) and test data (six kidneys), the classification quality of the models may be strongly influenced by the effect of outliers. Thus, the selection of the training and test dataset significantly determines the classification result. In addition, more data from nonfunctional kidneys can support classifier modeling to ensure future classification into three classes.

Moreover, in further studies, kidneys treated with NMP should also be re-transplanted after preservation. Thus, it could be determined whether inulin clearance, which was used as a reference for ex vivo renal function in this work, correlates with postoperative in vivo function. If it becomes apparent that inulin clearance is not suitable as a gold standard for ex vivo function assessment, the supervised learning methods presented here could be trained on other reference markers (e.g., histological markers). Thus, the approach presented here is highly adaptable depending on which reference markers are used for renal quality assessment.

### 4.6. Classification Based on the Preoperative Remuzzi Score

There are different approaches for histopathological evaluation of tissue biopsies in NMP experiments. A unified, standardized evaluation of biopsies to achieve comparability between different research groups has not yet been established. There are research groups that use the Remuzzi score or a modified Remuzzi score [21,42,62]. Other research groups base their histologic evaluation on very different biopsy parameters such as tubular injury, osmotic nephropathy, and hypokalemic nephropathy [34], or glomerular dilatation, tubular dilatation, and tubular necrosis [63].

Because there are a variety of scores for tissue biopsies of kidneys during NMP, we decided to use the Remuzzi score for our evaluation. To date, this score is the only one that has been compared to QAS in evaluating the condition of ex vivo kidneys during NMP [16].

In our study, a correlation between ex vivo function and histological damage based on the overall Remuzzi score or on the tubular atrophy parameter alone could not be detected (see Supplementary Material Figure S1: Classification based on the preoperative Remuzzi score; Table S5: Classification based on the tubular atrophy). However, it must be taken into account that the biopsies were taken from only one site of the organ, as is also common in transplantation. This therefore only allows a statement to be made about this small area of the kidneys and does not guarantee an overall assessment.

Because this is the first time that ex vivo renal function during the preservation phase has been correlated with renal histology, the results cannot be compared with the literature. However, it is well known that the use of histologic features for renal transplant acceptance and allocation criteria has limitations [5,6]. Firstly, these do not allow conclusions to be drawn about the prediction of graft survival. Secondly, histopathologic findings are based on subjective judgment, which leads to variability between pathologic results and thus limits the reliability of this analytical tool for predicting transplant outcome [5]. Objective methods such as those developed in this work could be used in the future to assess renal quality in addition to histological assessment.

## 5. Conclusions

In conclusion, the organ evaluation strategy presented here for assessing the functional status of normothermically perfused kidneys is based on supervised learning methods trained with blood and urine markers. For this purpose, a detailed significance analysis of the relationships of these markers to inulin clearance was performed. Significant markers were used as input to the classifiers, which subsequently provided acceptable classification performance for distinguishing kidneys according to their ex vivo renal function. In comparison, no correlation of the results of the Remuzzi Score with renal function could be found.

Our study demonstrates the feasibility of assigning kidneys according to their functional quality using classifiers based on blood- and urine-specific features. With the knowledge about the ex vivo functional quality of a kidney, organ evaluation strategies can be developed in the future, and thus be the first step for an appropriate organ assessment for kidney grafts from, e.g., marginal donors in the long term.

## Figures and Tables

**Table 1:** Overview of the functional classification of kidneys. Organs of the test dataset are marked with *. All kidneys without * are part of the trainings and validation dataset. In the column ‘Actual class’ the functional classes detected by gold standard are documented: non-functional (class 1, red), limited functional (class 2, yellow), functional (class 3, green). These results are contrasted with the classifier results. Crossed-out fields represent kidneys, which were not classified in the correct functional class.

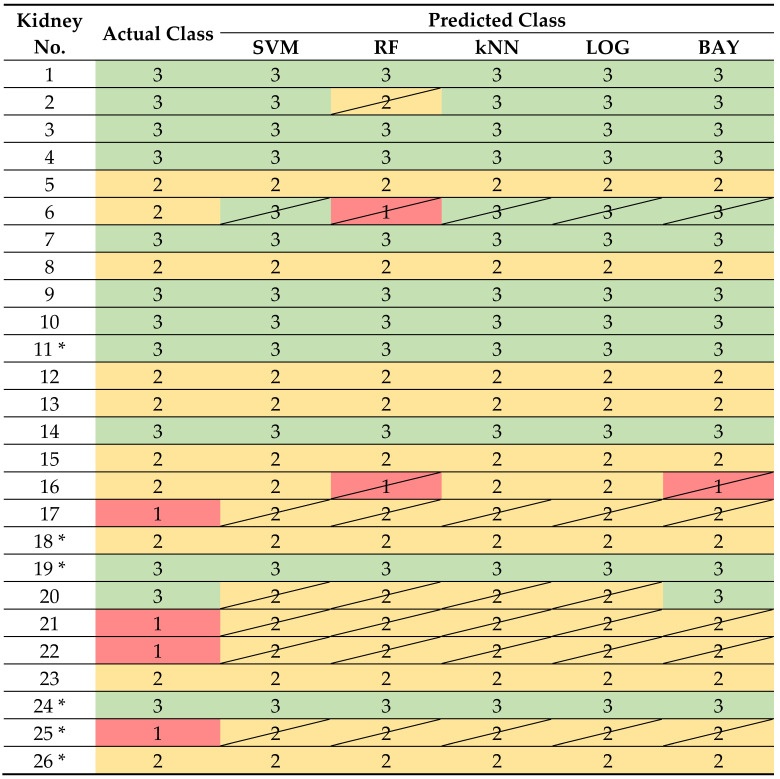

## Data Availability

All data will be made available upon reasonable request to the corresponding author.

## Supplementary Materials

The following supporting information can be downloaded at: https://www.mdpi.com/article/10.3390/biomedicines10123055/s1, Table S1: Characteristics of all kidneys; Table S2: Testing for multicollinearity; Table S3/S4: Functional classification based on blood and urine markers; Table S5: Classification based on the tubular atrophy; Figure S1: Classification based on the preoperative Remuzzi score.

## References

[1] Warmuzińska N., Łuczykowski K., Bojko B. (2022). A Review of Current and Emerging Trends in Donor Graft-Quality Assessment Techniques. J. Clin. Med..

[2] Branger P., Vogelaar S. (2021). Annual Report 2020.

[3] Rao P.S., Schaubel D.E., Guidinger M.K., Andreoni K.A., Wolfe R.A., Merion R.M., Port F.K., Sung R.S. (2009). A Comprehensive Risk Quantification Score for Deceased Donor Kidneys: The Kidney Donor Risk Index. Transplantation.

[4] Remuzzi G., Grinyò J., Ruggenenti P., Beatini M., Cole E.H., Milford E.L., Brenner B.M. (1999). Early Experience with Dual Kidney Transplantation in Adults using Expanded Donor Criteria. J. Am. Soc. Nephrol..

[5] Stallone G., Grandaliano G. (2019). To discard or not to discard: Transplantation and the art of scoring. Clin. Kidney J..

[6] Moeckli B., Sun P., Lazeyras F., Morel P., Moll S., Pascual M., Buhler L.H. (2019). Evaluation of donor kidneys prior to transplantation: An update of current and emerging methods. Transpl. Int..

[7] Kaths J.M., Paul A., Robinson L.A., Selzner M. (2018). Ex vivo machine perfusion for renal graft preservation. Transplant. Rev..

[8] Resch T., Cardini B., Oberhuber R., Weissenbacher A., Dumfarth J., Krapf C., Boesmueller C., Oefner D., Grimm M., Schneeberger S. (2020). Transplanting Marginal Organs in the Era of Modern Machine Perfusion and Advanced Organ Monitoring. Front. Immunol..

[9] De Beule J., Jochmans I. (2020). Kidney Perfusion as an Organ Quality Assessment Tool—Are We Counting Our Chickens Before They Have Hatched?. J. Clin. Med..

[10] Hamelink T.L., Ogurlu B., De Beule J., Lantinga V.A., Pool M.B.F., Venema L.H., Leuvenink H.G.D., Jochmans I., Moers C. (2022). Renal Normothermic Machine Perfusion: The Road Toward Clinical Implementation of a Promising Pretransplant Organ Assessment Tool. Transplantation.

[11] Markgraf W., Janssen M., Lilienthal J., Feistel P., Thiele C., Stöckle M., Malberg H. (2018). Hyperspectral imaging for ex-vivo organ characterization during normothermic machine perfusion. Eur. Urol. Suppl..

[12] Markgraf W., Lilienthal J., Feistel P., Thiele C., Malberg H. (2020). Algorithm for Mapping Kidney Tissue Water Content during Normothermic Machine Perfusion Using Hyperspectral Imaging. Algorithms.

[13] Markgraf W., Feistel P., Thiele C., Malberg H. (2018). Algorithms for mapping kidney tissue oxygenation during normothermic machine perfusion using hyperspectral imaging. Biomed. Eng. Biomed. Tech..

[14] Markgraf W., Lilienthal J., Feistel P., Mühle R., Thiele C., Malberg H., Janssen M. (2020). Hyperspectral imaging of porcine kidneys during normothermic ex vivo perfusion—An analysis of tissue-related renal ischemia injury. Transpl. Int..

[15] Schutter R., Lantinga V.A., Hamelink T.L., Pool M.B.F., Varsseveld O.C., Potze J.H., Hillebrands J., Heuvel M.C.V.D., Dierckx R.A.J.O., Leuvenink H.G.D. (2021). Magnetic resonance imaging assessment of renal flow distribution patterns during ex vivo normothermic machine perfusion in porcine and human kidneys. Transpl. Int..

[16] Hosgood S., Nicholson M.L. (2017). An Assessment of Urinary Biomarkers in a Series of Declined Human Kidneys Measured During Ex Vivo Normothermic Kidney Perfusion. Transplantation.

[17] Kaths J.M., Hamar M., Echeverri J., Linares I., Urbanellis P., Cen J.Y., Ganesh S., Dingwell L., Yip P., John R. (2017). Normothermic ex vivo kidney perfusion for graft quality assessment prior to transplantation. Am. J. Transplant..

[18] Wang L., Thompson E., Bates L., Pither T.L., Hosgood S.A., Nicholson M.L., Watson C.J., Wilson C., Fisher A.J., Ali S. (2020). Flavin Mononucleotide as a Biomarker of Organ Quality—A Pilot Study. Transplant. Direct.

[19] Woud W.W., Merino A., Hoogduijn M.J., Boer K., Hoogen M.W.F.V.D., Baan C.C., Minnee R.C. (2019). Nanoparticle Release by Extended Criteria Donor Kidneys During Normothermic Machine Perfusion. Transplantation.

[20] Hosgood S.A., Barlow A.D., Hunter J.P., Nicholson M.L. (2015). Ex vivo normothermic perfusion for quality assessment of marginal donor kidney transplants. Br. J. Surg..

[21] Hosgood S.A., Thompson E., Moore T., Wilson C.H., Nicholson M.L. (2017). Normothermic machine perfusion for the assessment and transplantation of declined human kidneys from donation after circulatory death donors. Br. J. Surg..

[22] Hosgood S.A., Saeb-Parsy K., Hamed M.O., Nicholson M.L. (2016). Successful Transplantation of Human Kidneys Deemed Untransplantable but Resuscitated by *Ex Vivo* Normothermic Machine Perfusion. Am. J. Transplant..

[23] Markgraf W., Mühle R., Lilienthal J., Kromnik S., Thiele C., Malberg H., Janssen M., Putz J. (2021). Inulin Clearance During Ex vivo Normothermic Machine Perfusion as a Marker of Renal Function. ASAIO J..

[24] Sommer F., Sun B., Fischer J., Goldammer M., Thiele C., Malberg H., Markgraf W. (2022). Hyperspectral Imaging during Normothermic Machine Perfusion—A Functional Classification of Ex Vivo Kidneys Based on Convolutional Neural Networks. Biomedicines.

[25] Takala J., Gullo A. (1997). Oxygen Consumption and Carbon Dioxide Production: Physiological Basis and Practical Application in Intensive Care. Anaesthesia, Pain, Intensive Care and Emergency Medicine.

[26] Bizouarn P., Soulard D., Blanloeil Y., Guillet A., Goarin Y. (1992). Oxygen consumption after cardiac surgery —A comparison between calculation by Fick’s principle and measurement by indirect calorimetry. Intensiv. Care Med..

[27] de Boode W.P., Hopman J.C.W., Daniëls O., van der Hoeven H.G., Liem K.D. (2007). Cardiac Output Measurement Using a Modified Carbon Dioxide Fick Method: A Validation Study in Ventilated Lambs. Pediatr. Res..

[28] Douglas A.R., Jones N.L., Reed J.W. (1988). Calculation of whole blood CO2 content. J. Appl. Physiol..

[29] Belsley E., Kuh E., Welsch R.E. (1980). Regression Diagnostics: Identifying Influential Data and Sources of Collinearity.

[30] Frochte J. (2019). Maschinelles Lernen: Grundlagen und Algorithmen in Python.

[31] BenSaïda A. Shapiro-Wilk and Shapiro-Francia Normality Tests Version 1.1.0.0 2019. https://www.mathworks.com/matlabcentral/fileexchange/13964-shapiro-wilk-and-shapiro-francia-normality-tests.

[32] Trujillo-Ortiz A. Levenetest Version 1.0.0.0 2019. https://www.mathworks.com/matlabcentral/fileexchange/3375-levenetest?s%7B%5C_%7Dtid=srchtitle.

[33] Trujillo-Ortiz A. Welchanova Version 1.1.0.0 2019. https://www.mathworks.com/matlabcentral/fileexchange/37121-welchanova?s%7B%5C_%7Dtid=srchtitle.

[34] Trujillo-Ortiz A. GHtest Version 1.0.0.0 2019. https://www.mathworks.com/matlabcentral/fileexchange/3676-ghest?s%7B%5C_%7Dtid=srchtitle.

[35] Fabry G., Doorschodt B.M., Grzanna T., Boor P., Elliott A., Stollenwerk A., Tolba R.H., Rossaint R., Bleilevens C. (2019). Cold Preflush of Porcine Kidney Grafts Prior to Normothermic Machine Perfusion Aggravates Ischemia Reperfusion Injury. Sci. Rep..

[36] Smith S.F., Adams T., Hosgood S.A., Nicholson M.L. (2017). The administration of argon during ex vivo normothermic perfusion in an experimental model of kidney ischemia–reperfusion injury. J. Surg. Res..

[37] Hosgood S.A., Moore T., Kleverlaan T., Adams T., Nicholson M.L. (2017). Haemoadsorption reduces the inflammatory response and improves blood flow during ex vivo renal perfusion in an experimental model. J. Transl. Med..

[38] Maassen H., Hendriks K.D.W., Venema L.H., Henning R.H., Hofker S.H., Van Goor H., Leuvenink H.G.D., Coester A.M. (2019). Hydrogen sulphide-induced hypometabolism in human-sized porcine kidneys. PLoS ONE.

[39] Hosgood S.A., Moore T., Qurashi M., Adams T., Nicholson M.L. (2018). Hydrogen Gas Does Not Ameliorate Renal Ischemia Reperfusion Injury in a Preclinical Model. Artif. Organs.

[40] Yang C., Hosgood S.A., Meeta P., Long Y., Zhu T., Nicholson M.L., Yang B. (2015). Cyclic Helix B Peptide in Preservation Solution and Autologous Blood Perfusate Ameliorates Ischemia-Reperfusion Injury in Isolated Porcine Kidneys. Transplant. Direct.

[41] A Hosgood S., Yates P.J., Nicholson M.L. (2014). 1400W reduces ischemia reperfusion injury in an ex-vivo porcine model of the donation after circulatory death kidney donor. World J. Transplant..

[42] Adams T., Hosgood S.A., Nicholson M.L. (2019). Physiological effects of altering oxygenation during kidney normothermic machine perfusion. Am. J. Physiol. Physiol..

[43] Aburawi M.M., Fontan F.M., Karimian N., Eymard C., Cronin S., Pendexter C., Nagpal S., Banik P., Ozer S., Mahboub P. (2019). Synthetic hemoglobin-based oxygen carriers are an acceptable alternative for packed red blood cells in normothermic kidney perfusion. Am. J. Transplant..

[44] Adams T., Patel M., Hosgood S.A., Nicholson M.L. (2017). Lowering Perfusate Temperature From 37 °C to 32 °C Diminishes Function in a Porcine Model of Ex Vivo Kidney Perfusion. Transplant. Direct.

[45] Patel M., Hosgood S., Nicholson M.L. (2014). The effects of arterial pressure during normothermic kidney perfusion. J. Surg. Res..

[46] Hendriks K.D.W., Brüggenwirth I.M.A., Maassen H., Gerding A., Bakker B., Porte R.J., Henning R.H., Leuvenink H.G.D. (2019). Renal temperature reduction progressively favors mitochondrial ROS production over respiration in hypothermic kidney preservation. J. Transl. Med..

[47] Mancina E., Kalenski J., Paschenda P., Beckers C., Bleilevens C., Boor P., Doorschodt B.M., Tolba R.H. (2015). Determination of the Preferred Conditions for the Isolated Perfusion of Porcine Kidneys. Eur. Surg. Res..

[48] Blum M.F., Liu Q., Soliman B., Dreher P., Okamoto T., Poggio E.D., Goldfarb D.A., Baldwin W.M., Quintini C. (2017). Comparison of normothermic and hypothermic perfusion in porcine kidneys donated after cardiac death. J. Surg. Res..

[49] Georgiades F., Hosgood S.A., Butler A.J., Nicholson M.L. (2019). Use of ex vivo normothermic machine perfusion after normothermic regional perfusion to salvage a poorly perfused DCD kidney. Am. J. Transplant..

[50] Hosgood S.A., Patel M., Nicholson M.L. (2013). The conditioning effect of ex vivo normothermic perfusion in an experimental kidney model. J. Surg. Res..

[51] Hosgood S.A., Barlow A.D., Dormer J., Nicholson M.L. (2015). The use of ex-vivo normothermic perfusion for the resuscitation and assessment of human kidneys discarded because of inadequate in situ perfusion. J. Transl. Med..

[52] Chandak P., Phillips B., Uwechue R., Thompson E., Bates L., Ibrahim I., Sewpaul A., Figueiredo R., Olsburgh J., Hosgood S. (2019). Dissemination of a novel organ perfusion technique: Ex vivo normothermic perfusion of deceased donor kidneys. Artif. Organs.

[53] Jochmans I., Moers C., Smits J.M., Leuvenink H.G.D., Treckmann J., Paul A., Rahmel A., Squifflet J.-P., Van Heurn E., Monbaliu D. (2011). The Prognostic Value of Renal Resistance During Hypothermic Machine Perfusion of Deceased Donor Kidneys. Am. J. Transplant..

[54] Yang B., Hosgood S., Da Z., Harper S.J., Waller H.L., Kay M.D., Furness P.N., Nicholson M.L. (2012). Biomarkers assessing warm ischemic injury using an isolated porcine kidney hemoreperfusion model. Exp. Biol. Med..

[55] A Hosgood S., Saeb-Parsy K., Wilson C., Callaghan C., Collett D., Nicholson M.L. (2017). Protocol of a randomised controlled, open-label trial of ex vivo normothermic perfusion versus static cold storage in donation after circulatory death renal transplantation. BMJ Open.

[56] Kaths J.M., Spetzler V.N., Goldaracena N., Echeverri J., Louis K.S., Foltys D.B., Strempel M., Yip P., John R., Mucsi I. (2015). Normothermic Ex Vivo Kidney Perfusion for the Preservation of Kidney Grafts prior to Transplantation. J. Vis. Exp..

[57] Hosgood S., Harper S., Kay M., Bagul A., Waller H., Nicholson M.L. (2006). Effects of arterial pressure in an experimental isolated haemoperfused porcine kidney preservation system. Br. J. Surg..

[58] van Essen G.J., Hekkert M.T.L., Sorop O., Heinonen I., van der Velden J., Merkus D., Duncker D.J. (2018). Cardiovascular Function of Modern Pigs Does not Comply with Allometric Scaling Laws. Sci. Rep..

[59] Waller H.L., Harper S.J.F., Hosgood S.A., Bagul A., Yang B., Kay M.D., Kaushik M., Nicholson M.L. (2006). Biomarkers of oxidative damage to predict ischaemia-reperfusion injury in an isolated organ perfusion model of the transplanted kidney. Free Radic. Res..

[60] Bhangoo R.S., Hall I.E., Reese P.P., Parikh C.R. (2012). Deceased-donor kidney perfusate and urine biomarkers for kidney allograft outcomes: A systematic review. Nephrol. Dial. Transplant..

[61] He X., Chen G., Zhu Z., Zhang Z., Yuan X., Han M., Zhao Q., Zheng Y., Tang Y., Huang S. (2019). The First Case of Ischemia-Free Kidney Transplantation in Humans. Front. Med..

[62] Weissenbacher A., Faro M.L.L., Boubriak O., Soares M.F., Roberts I.S., Hunter J.P., Voyce D., Mikov N., Cook A., Ploeg R.J. (2019). Twenty-four–hour normothermic perfusion of discarded human kidneys with urine recirculation. Am. J. Transplant..

[63] Pool M.B.F., Hartveld L., Leuvenink H., Moers C. (2020). Normothermic machine perfusion of ischaemically damaged porcine kidneys with autologous, allogeneic porcine and human red blood cells. PLoS ONE.

