# Supplementary material for: Preoperative Function Assessment of Ex Vivo Kidneys with Supervised Machine Learning Based on Blood and Urine Markers Measured during Normothermic Machine Perfusion
Source: Biomedicines. 2022 Nov 28;10(12):3055. doi: 10.3390/biomedicines10123055 (PMC9776285; doi:10.3390/biomedicines10123055)
Supplement: Supplementary file 1 [file biomedicines-10-03055-s001.zip › Supplement_Digital_Material_Table_S1.pdf]

Supplementary Material – S1

Characteristics of all kidneys

Table S1. Overview of kidneys preserved ex vivo with normothermic machine perfusion (NMP). Listed are the sex, race, warm ischemia time, cold ischemia time, glomerular filtration rate (GFR) and inulin eliminated from the blood during NMP ($I_{e,total}$) for all kidneys of a class, kidneys in the training and validation data set, and kidneys in the test data set. Data are presented as mean ± standard deviation. Previously published in [24].

Pig and Kidney Characteristics	Class 1	Class 2	Class 3
Overall	*n* = 4	*n* = 10	*n* = 12
Female:Male	3:1	4:6	4:8
German Landrace:Swabian Hall	3:1	4:6	4:8
Warm ischemia time in min	122 ± 52	67 ± 30	12 ± 7
Cold ischemia time in min	404 ± 246	402 ± 201	312 ± 170
GFR in mL/min/100 g	1.3 ± 0.6	3.0 ± 0.9	14.8 ± 10.1
$I_{e,total}$ in %	45 ± 1	72 ± 9	97 ± 3
Training and validation	*n = 3*	*n = 8*	*n = 9*
Female:Male	2:1	3:5	2:7
German Landrace:Swabian Hall	2:1	3:5	2:7
Warm ischemia time in min	114 ± 61	66 ± 34	12 ± 7
Cold ischemia time in min	341 ± 259	397 ± 233	305 ± 125
GFR in mL/min/100 g	1.5 ± 0.8	2.9 ± 0.8	16.4 ± 10.4
$I_{e,total}$ in %	45 ± 1	72 ± 9	97 ± 3
Test	*n = 1*	*n = 2*	*n = 3*
Female:Male	1:0	1:1	2:1
German Landrace:Swabian Hall	1:0	1:1	2:1
Warm ischemia time in min	145	73 ± 10	13 ± 8
Cold ischemia time in min	594	420 ± 121	333 ± 309
GFR in mL/min/100 g	0.8	3.4 ± 1.4	10.0 ± 9.3
$I_{e,total}$ in %	44	75 ± 12	98 ± 1

[24] Sommer, F.; Sun, B.; Fischer, J.; Goldammer, M.; Thiele, C.; Malberg, H.; Markgraf, W. Hyperspectral Imaging during Normothermic Machine Perfusion-A Functional Classification of Ex Vivo Kidneys Based on Convolutional Neural Networks. Biomedicines 2022, 10(2), 397. https://doi.org/10.3390/biomedicines10020397. This article is an open access article distributed under the terms and conditions of the Creative Commons Attribution (CC BY) license (https://creativecommons.org/licenses/by/4.0/).
