# Supplementary material for: Preoperative Function Assessment of Ex Vivo Kidneys with Supervised Machine Learning Based on Blood and Urine Markers Measured during Normothermic Machine Perfusion
Source: Biomedicines. 2022 Nov 28;10(12):3055. doi: 10.3390/biomedicines10123055 (PMC9776285; doi:10.3390/biomedicines10123055)
Supplement: Supplementary file 1 [file biomedicines-10-03055-s001.zip › Supplement_Digital_Material_Table_S2.pdf]

Supplementary Material – Table S2

Testing for multicollinearity

Table S2. Detection of multicollinearity using VIF. First, all 11 markers were tested for multicollinearity (VIF_{total}). Due to critical correlation (VIF > 10), two markers were excluded. The remaining 9 markers were retested for multicollinearity ($VIF_{reduced}$).

Marker	VIF_{total}	$VIF_{reduced}$
RBF	6.2	2.7
AP	8.1	1.7
T_{Kidney}	1.7	1.2
IRR	13.6	X
VO_2	15.3	1.8
DO_2	22.0	X
ERO_2	1.1	1.2
CO_2P	1.1	1.0
Urine production	2.5	2.3
Relative urea concentration	2.2	2.1
Relative GST activity	1.1	1.1

VIF = 1
1 < VIF ≤ 5
5 < VIF ≤ 10
VIF > 10
