# Supplementary material for: Preoperative Function Assessment of Ex Vivo Kidneys with Supervised Machine Learning Based on Blood and Urine Markers Measured during Normothermic Machine Perfusion
Source: Biomedicines. 2022 Nov 28;10(12):3055. doi: 10.3390/biomedicines10123055 (PMC9776285; doi:10.3390/biomedicines10123055)
Supplement: Supplementary file 1 [file biomedicines-10-03055-s001.zip › Supplement_Digital_Material_Table_S3_S4.pdf]

Supplementary Material – Table S3/S4

Functional classification based on blood and urine markers

Table S3. Goodness of the classifiers on the validation dataset for the division of kidneys into three classes. The following variants were investigated: A) parameters where significant differences were found between classes, and all measurement time points up to 240 min (56 features), parameters as well as their measurement time points up to B) 240 min (38 features), C) 180 min (27 features), D) 120 min (16 features), E) 60 min (7 features) where significant differences were found between classes.

Variant	SVM			RF			kNN			LOG			BAY		
	Acc.	Rec.	Prec.	Acc.	Rec.	Prec.	Acc.	Rec.	Prec.	Acc.	Rec.	Prec.	Acc.	Rec.	Prec.
A	0.7	0.7	0.7	0.6	0.6	0.6	0.7	0.7	0.7	0.75	0.75	0.75	0.7	0.7	0.7
B	0.7	0.7	0.7	0.7	0.7	0.7	0.75	0.75	0.75	0.75	0.75	0.75	0.75	0.75	0.75
C	0.75	0.75	0.75	0.65	0.65	0.65	0.75	0.75	0.75	0.75	0.75	0.75	0.75	0.75	0.75
D	0.65	0.65	0.65	0.6	0.6	0.6	0.65	0.65	0.65	0.75	0.75	0.75	0.55	0.55	0.55
E	0.6	0.6	0.6	0.6	0.6	0.6	0.5	0.5	0.5	0.65	0.65	0.65	0.55	0.55	0.55

Table S4. Goodness of the classifiers on the test dataset for the division of kidneys into three classes. The following variants were investigated: A) parameters where significant differences were found between classes, and all measurement time points up to 240 min (56 features), parameters as well as their measurement time points up to B) 240 min (38 features), C) 180 min (27 features), D) 120 min (16 features), E) 60 min (7 features) where significant differences were found between classes.

Variant	SVM			RF			kNN			LOG			BAY		
	Acc.	Rec.	Prec.	Acc.	Rec.	Prec.	Acc.	Rec.	Prec.	Acc.	Rec.	Prec.	Acc.	Rec.	Prec.
A	0.83	0.83	0.72	0.67	0.67	0.67	0.67	0.67	0.67	0.83	0.83	0.72	0.83	0.83	0.72
B	0.83	0.83	0.72	0.67	0.67	0.67	0.67	0.67	0.67	0.83	0.83	0.72	0.83	0.83	0.72
C	0.83	0.83	0.72	0.67	0.67	0.67	0.83	0.83	0.72	0.83	0.83	0.72	0.83	0.83	0.72
D	0.83	0.83	0.72	0.83	0.83	0.72	0.67	0.67	0.67	0.83	0.83	0.72	0.83	0.83	0.72
E	0.67	0.67	0.67	0.5	0.5	0.44	0.5	0.5	0.61	0.67	0.67	0.67	0.67	0.67	0.67
