# Supplementary material for: Preoperative Function Assessment of Ex Vivo Kidneys with Supervised Machine Learning Based on Blood and Urine Markers Measured during Normothermic Machine Perfusion
Source: Biomedicines. 2022 Nov 28;10(12):3055. doi: 10.3390/biomedicines10123055 (PMC9776285; doi:10.3390/biomedicines10123055)
Supplement: Supplementary file 1 [file biomedicines-10-03055-s001.zip › Supplement_Digital_Material_Table_S5_Figure_S1.pdf]

Supplementary Material – Table S5/ Figure S1

Classification based on the preoperative Remuzzi score

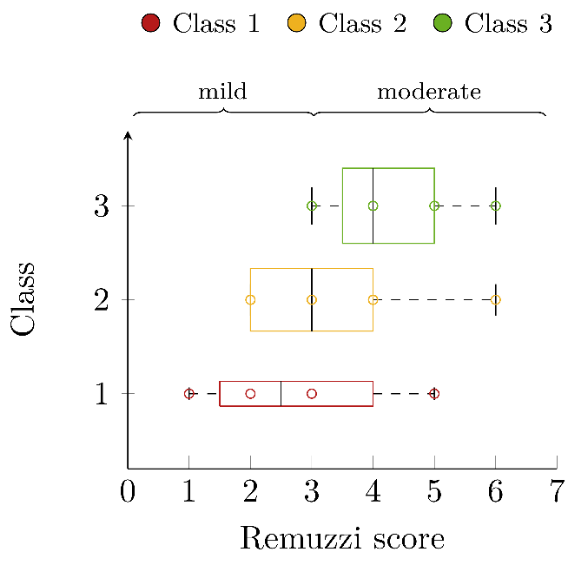

Figure S1. Results of histological findings. The correlation of functional class with Remuzzi score is shown. n = 4 for nonfunctional kidneys (class 1), n = 10 for kidneys with limited function (class 2), n = 12 for functional kidneys (class 3).

Classification based on the tubular atrophy

Table S5: Results of the histological findings with regard to the acute damage. The relation between functional class and tubular atrophy, a parameter of the Remuzzi Score, is shown. Histologic findings demonstrated tubular atrophy values of 0, 1+, 2+, but never 3+. n = 4 for nonfunctional kidneys (class 1), n = 10 for kidneys with limited function (class 2), n = 12 for functional kidneys (class 3).

	**Evaluation of tubular atrophy according to Remuzzi [4]**		
**Class**	**0**	**1+**	**2+**
**1**	2	2	0
**2**	4	5	1
**3**	1	7	4

[4] Remuzzi, G.; Grinyò, J.; Ruggenenti, P.; Beatini, M.; Cole, E.H.; Milford, E.L.; Brenner, B.M. Early experience with dual kidney transplantation in adults using expanded donor criteria. *JASN* **1999**, *10*, 2591–2598. https://doi.org/10.1681/ASN.V10122591.
